# Current News and Opinion

**Published:** 1884-05

**Authors:** 


### Current News and Opinion.

### "TOO BUSY."

The "Busy Practitioner" humbug is handled by *Gaillard's Medical Monthly Journal* in the outspoken manner characteristic of its editor: "The most widespread and at the same time the most transparent adulation is that offered by so many of the smaller journals of the profession, in connection with the subject of the 'busy practitioner' and 'long-winded articles.' As to the 'busy practitioners' not having time to read 'long-winded articles,' such a statement is manifest buncombe and silly flattery, offered to the foolish among the profession by such journals as have not the space in which to offer, or ability with which to appreciate, carefully elaborated papers. There is no physician who is too busy to read good papers. Indeed, if he is very busy he is so because he has been a careful student of such literature. It is only to the true men of the profession that reference is here made, and these men, if busy, are busy, not because of having read miscellaneous paragraphs and foolish formulas, cut from medical briefs and almanacs, but because of their study of the best papers of the best men. The reader who turns from the paper of a writer because it is long is on the high and brief road to idleness, worthlessness and ruin. He has mistaken his vocation, and the sooner he gives it up the better it will be, not only for himself but his patients, and above all, his brethren. He is already a drone in the hive. When one reads, as he does every day in the smaller journals, the statement made to the average practitioner throughout the United States, the doctors of eight or ten patients daily, and many of them even less, that they are too busy to read long articles, but must read the little paragraphs (in such periodicals) as to what is 'good for' something, what is he to do but smile? But if he has the misfortune to be an editor, there is one other thing he can do, must do—it is to expose the silly fraud herein mentioned, and to help men see the truth."—*Weekly Medical Review.*

The above extract from the medical press is quite as applicable to dentists. Who are the practitioners that are "too busy" to read their professional journals? Almost invariably obscure men, cheap dentists, who vainly desire to impress others with the magnitude of their practice. The really busy men are those who realize that an acquaintance with professional literature is a necessity; who have built up a remunerative business through their intelligence and acquaintance with the latest methods, obtained only by a careful study of the journals. Go into any city or town and inquire who has the largest practice, and it will invariably be found that the fortunate man is a subscriber to and a reader of all the best dental journals. It is only the pretenders, the make-believes, the *putterers*, and the Cheap Johns, who are "too busy" for study.

---

### THE THERAPEUTIC APPLICATION OF NITROUS OXIDE GAS.

From a series of experiments made in Prof. Botkin's laboratory in St. Petersburg, Dr. S. Klikowitsch (Virchows Archives xliv, 2), draws the following conclusions:

1. Nitrous oxide gas is incapable of supporting respiration in animals and plants, and, like other indifferent gases, leads to death from asphyxia. The asphyxia produced by this gas, however, presents points of contrast to the asphyxia produced by other means.

2. Nitrous oxide gas produces no chemical or morphological changes in the blood of animals, but is dissolved in it and again eliminated, according to physical laws, without apparently being broken up into nitrogen and oxygen.

3. Anæsthesia with laughing gas is so closely associated with insufficient oxidation of the blood, that it cannot be regarded as absolutely without danger, especially in diseases of the heart, lungs, or blood-vessels.

4. The association of laughing gas with twenty per cent. of oxygen completely removes the possibility of asphyxia, and produces a number of results capable of therapeutic application.

5. Under the influence of the mixture of laughing gas and twenty per cent. of oxygen, in the majority of healthy subjects, the heart's pulsations are increased, the pulse-wave diminished, and the respiratory movements decreased in number and increased in depth; these effects pass off in from three to five minutes.

6. In six cases of weak heart action, the above gaseous mixture produced no unfavorable results; on the other hand, the pulse was decreased in frequency and increased in strength. These effects lasted from one to two hours.

7. In cases of disturbed respiratory innervation the mixture of laughing gas and oxygen regulated the respiratory rhythm and rapidly removed the subjective and objective signs of insufficient oxidation of the blood.

8. This gaseous mixture acts as a transient anæsthetic, and in angina pectoris causes a rapid removal of suffering.

9. It is preferable to chloroform as an anæsthetic in labor.

10. Vomiting and cough of reflex origin are arrested by a few inhalations of this mixture of gases.—*Phil. Med. Times.*

---

### ILLINOIS STATE DENTAL SOCIETY.

The twentieth annual meeting will be held in the Senate Chamber, Springfield, commencing Tuesday, May 13th, and continuing four days.

### SUBJECTS AND ESSAYISTS.

I.—Exposed Dental Pulps and their Treatment. S. F. Duncan, Wilmington.
Discussion opened by J. N. Crouse, Chicago.

II.—Reflex Pain. . . . . K. C. Moody, Mendota.
Discussion opened by T. L. Gilmer, Quincy.

III.—Origin of the Defective Tooth Structure, Known as Pitted, Furrowed, or Cribriform Enamel. . . W. H. Eames, St. Louis, Mo.
Discussion opened by L. C. Ingersoll, Keokuk.

IV.—Inflammation. . . . . Homer Judd, Alton.
Discussion opened by W. B. Woodward, Peoria.

V.—Chemistry. . . . Wm. H. Taggart, Freeport.
Discussion opened by E. S. Talbot, Chicago.

VI.—Dental Education, past, present and future. A. H. Fuller, St. Louis, Mo.
Discussion opened by Geo. H. Cushing, Chicago.

VII.—Dental-Embryonal Histology. . Will X. Sudduth, Bloomington.
Discussion opened by A. W. Harlan, Chicago.

VIII.—Some Applications of Electrolysis in Dentistry. W. B. Ames, Chicago.
Discussion opened by T. W. Brophy, Chicago.

IX.—Irregularities in Human Teeth—Treatment and Cause.
J. J. R. Patrick, Belleville.
Discussion opened by W. N. Morrison, St. Louis.

X.—Life as a Force. . . G. V. Black, Jacksonville.
Discussion opened by J. Taft, Cincinnati.

XI.—Illinois State Dental Society—What has it Accomplished?
C. R. E. Koch, Chicago.
Discussion opened by C. A. Kitchen, Rockford.

THURSDAY MORNING.—CLINICAL OPERATORS.

1.—Edgar D. Swain, Chicago. (Gold Crown.)
2.—H. H. Townsend, Pontiac. (Root Filling.)
3.—W. N. Conrad, St. Louis. (Porcelain Crown.)
4.—E. Honsinger, Chicago. (Filling with Crystal Gold.)
5.—C. F. Matteson, Chicago. (Gold Filling.)
6.—W. T. Magill, Rock Island. (Gold Filling)
7.—J. A. Swasey, Chicago. (Filling with Gold and Tin.)
8.—H. J. McKellops, St. Louis. (Gold Filling, Using Bonwill's Engine Plugger.)
9.—J. G. Reid. (Filling Roots with Gutta Percha.)
10.—S. M. Sturgis, Quincy. (Gold Plate, with Rubber Attachment.)

EDMUND NOYES, Chicago, President.
J. W. WASSALL, Chicago, Secretary.

---

### KANSAS STATE DENTAL SOCIETY

The thirteenth annual meeting of the Kansas State Dental Association will be held at Hiawatha, Kansas, commencing on Tuesday, May 6th. This meeting is held at the same time and place as the annual meeting of the Nebraska State Association, in accordance with the unanimous desire of the two Associations for a joint meeting.

Essays will be read by members of the Kansas Association, as follows:

The Treatment of Dental Diseases in Childhood and Youth,
DR. A. H. THOMPSON, Topeka.
Gold and Aluminum vs. Rubber for Artificial Dentures,
DR. L. M. MATTHEWS, Fort Scott.
Treatment of Alveolar Abscess and Root Filling,
DR. W. H. SHULZE, Atchison.
Prevention of Decay, . . DR. W. M. SHIRLEY, Hiawatha.
Tobacco, . . . . DR. J. A. YOUNG, Emporia.
Dental Students, . . . DR. L. P. MEREDITH, Abilene.
Exposed Nerves, . . DR. J. D. PATTERSON, Kansas City.

Arrangements have been made for a proper division of the time, and for joint discussion of all the scientific and literary programme of both Associations. J. D. PATTERSON, Secretary.
Kansas City, Mo.

A. M. CALLAHAN, Topeka, President.

### DENTAL MEETINGS FOR MAY.

The following societies hold meetings during the month of May:

Am. Med. Association—First Tuesday, Washington, D. C.
California State—Third Tuesday, San Francisco.
Connecticut State—Third Tuesday, at Hartford.
Georgia State—Second Tuesday, at Atlanta.
Illinois State—Second Tuesday, at Decatur.
Iowa State—First Tuesday, at Council Bluff.
Kansas State—First Tuesday, }
Nebraska State—First Tuesday, } Hiawatha, Kan., in joint session.
Minnesota State—Fourth Tuesday, at Winona.
South Carolina State—First Tuesday at Cheraw.
New York State—Second Wednesday, at Albany.
Mad River Valley—Third Tuesday, at Dayton, Ohio.
Southern—First Tuesday, at Lexington, Kentucky.
Northern Ohio—Second Tuesday, at Cleveland, O.
Odontographic—(Penn.)—First Wednesday, at Philadelphia.
Odontological—(N. Y.)—Third Tuesday, at New York.
First District—(N. Y.)—First Tuesday, at New York.
Sixth District—(N. Y.)—First Tuesday, at Binghamton.

---

### DENTAL SOCIETY OF THE STATE OF NEW YORK.

The Dental Society of the State of New York will hold its sixteenth annual meeting at Albany, Wednesday and Thursday, May 14 and 15, 1884.

PROGRAMME.

President's Address, . . . L. S. Straw, Newburgh.
Histology (to be Illustrated by Diagrams, etc.), Frank Abbott, New York.
The Influence of Antiseptics, Filling Materials, etc., upon the Fungi of Dental Caries (to be read by W. C. Barrett), . W. D. Miller, Berlin.
That Will Do, . . . J. G. Ambler, New York.
The Past, Present, and Future of Dentistry, E. Parmly Brown, Flushing.
Preparation of the Mouth for Artificial Teeth, W. H. Atkinson, New York.
On the Transposition of Certain Functions of the Teeth, }
Anatomical Reasons for Dento-Alveolar Abscess of the Hard-Palate, }
J. Edw. Line, Rochester.
Incidents of Office Practice, . Members and Privileged Visitors.

J. Edw. Line, D. D. S., Rochester, N. Y.,
Secretary.

### PERPETUAL INJUNCTION.

In the U. S. Circuit Court in Maryland, it was, on the 10th of March, 1884, adjudged and decreed that a perpetual injunction be issued against Louis E. Wetter, and eighteen others, restraining them from imitating the labels of the Rumford Chemical Works, manufacturers of Horsford's Baking Powder, and also from using their old bottles.

The defendants were required to bring into court all fraudulent labels, and all imitation powder, for destruction.

It was decreed that the Rumford Chemical Works be entitled to receive the profits which have been diverted from it by reason of the infringement, and the defendants were ordered to pay all costs.

Thus is another victory scored for the Rumford Chemical Works, who, not long since, caused several parties to be heavily fined for violating the injunction of the Supreme Court, restraining all persons from offering for sale "Acid Phosphate" (so called), in any package which shall be a substantial or colorable imitation of Horsford's Acid Phosphate.

---

### AMERICAN MEDICAL ASSOCIATION.

President—Dr. Austin Flint, Sen., New York.

Secretary—Dr. Wm. B. Atkinson, Philadelphia.

### SECTION OF ORAL AND DENTAL SURGERY.

Chairman—Dr. T. W. Brophy, Chicago.

Secretary—Dr. J. S. Marshall, Chicago.

Meets at Washington, D. C., Tuesday, Wednesday and Thursday, May 6th, 7th and 8th, 1884.

---

### A CASE OE ŒSOPHAGOTOMY.

In the April number of *The American Journal of the Medical Sciences*, Dr. Louis A. La Garde reports a successful case of this rare operation (the opening of the œsophagus), for the dislodgment of a plate of artificial teeth, and he shows that existing statistics do not support the view of Sir William Fergusson and Nelaton, that œsophagotomy is a dangerous operation.

### MICHIGAN DENTAL ASSOCIATION.

A condensed report of the annual meeting at Detroit is crowded out of this number. The officers elected for the ensuing year are :

President—Dr. J. Lathrop, Detroit.

First Vice President—Dr. F. W. Clawson, Detroit.

Second Vice Pesident—Dr. E. C. Moore, Detroit.

Secretary—Dr J. B. McGregor, Port Huron.

Treasurer—Dr. H. K. Lathrop, Detroit.

A resolution was passed that the calling of a mixture of nitrous oxide gas and the vapor of chloroform or ether, "Vitalized Air," is a misnomer, and is calculated to deceive and mislead the public, and any member of the Association so advertising shall be deemed guilty of an infraction of the Code of Ethics.

---

### SIXTH DISTRICT DENTAL SOCIETY OF NEW YORK.

The Sixth District Dental Society will hold its fifteenth annual meeting in the Common Council Rooms at Binghamton, on Tuesday, May 6th, 1884, at 2 o'clock P. M. The president, Dr. M. D. Jewell, of Richfield Springs, will deliver the annual address. The following papers will be read:

Combination of Gold and Amalgam; Dr. F. B. Darby, of Elmira.

Iodoform; Dr. E. D. Downs, of Owego.

Volunteer essays are invited. All dentists residing in the district are cordially invited to be present.

E. D. DOWNS, Secretary.

---

### CICERO ON DENTISTRY.

*Editor Christian Advocate :*

Below find a quotation from Cicero on "The Laws." The "Twelve tables" from which it seems to be taken, were enacted about B. C. 450 : "And since in the law there was this clause, that gold should not be buried with the dead, how humane is the exception made by another law, *that if the teeth of the deceased were fastened with gold*, the corpse might be buried or burned without taking it away, and no wrong be done."—*The Microscope.*

### PERSONAL.

Our associate, Dr. C. F. W. Bodecker, will sail about May 20th for Europe, to be gone during the summer. He has earned a vacation by his long-continued and exhaustive labor, but if any one thinks that he goes abroad for a summer's idleness, he does not know Dr. Bodecker. The readers of the INDEPENDENT PRACTITIONER will benefit by the Doctor's study in Europe, for he will make it a special business to pick up anything that may interest them. He has the best wishes of his associates and many professional friends for a pleasant and profitable trip.

Dr. John D. Maynard has gone to Paris, France, where he will be associated with Dr. John W. Crane, at 41 Boulevard des Capucines.

---

### SECOND DISTRICT DENTAL SOCIETY.

At the annual meeting of the Second District Dental Society, held on March 11th, at the office of Dr. A. H. Brockway, Brooklyn, the following officers were elected:

President, J. H. Holly, Warwick; Vice-President, E. P. Brown, Flushing; Recording Secretary, A. N. Roussel, Brooklyn; Corresponding Secretary, E. T. Van Woert, Brooklyn; Treasurer, L. G. Wilder, Brooklyn; Librarian, F. W. Dolbeare, Brooklyn; Delegates to the State Society, Drs. E. H. Dickey and Mills; Censors, A. H. Brockway, Wm. Jarvie, Jr., O. E. Hill, H. G. Mirick, C. F. Allen.

A. N. ROUSSEL, D. D. S., Recording Secretary.

---

### MAD RIVER VALLEY DENTAL SOCIETY.

The Mad River Valley Dental Society will hold its annual meeting in the parlor of the Phillips House, Dayton, Tuesday, May 20, 1884. Sessions begin at 10 o'clock, A. M., 2 and 7 P. M.

### SUBJECTS FOR DISCUSSION.

1. The Value of Pulpless Teeth and Roots.
2. Inflammation.
3. Alveolar Abscess.
4. The Promotion of Osseous Development.
5. Facial Neuralgia.

President, A. Berry; Vice-President, C. M. Wright; Secretary and Treasurer, W. H. Sillito.

### NORTHERN OHIO DENTAL ASSOCIATION.

The twenty-fifth annual meeting will be held in Cleveland, Tuesday and Wednesday, May 13th and 14th, 1884, commencing at 10, A. M. Tuesday. Meeting to be held at Board of Education Rooms public library. A cordial invitation is extended to all the profession.

### SUBJECTS FOR DISCUSSION.

1. The Causes of Failure in Dental Operations.
2. Materials for Filling Teeth, Their Relative Value, and How to Use Them.
3. Prosthetic Dentistry, Including Artifical Crowns.
4. Subjects proposed by members, and quiz.

GALE FRENCH, President.

H. F. HARVEY, Cor. Sec.

---

### SOUTHERN DENTAL ASSOCIATION.

The sixteenth annual session will be held in Lexington, Ky., commencing May 6th and continuing three days. Reduction of fares upon the railroads, also special rates at the hotels, have been secured.

The Chairman of the Committee of Arrangements, Dr. A. O. Rawls of Lexington, proposes that the members and visitors shall be entertained in "Old Kentucky style," and the meeting will without doubt be a memorable one.

President, H. J. McKellops, St. Louis, Mo.

Corresponding Secretary—J. P. Holmes, Macon, Ga.

Rec. Sec.—W. H. Hoffman, Gastonia, N. C.

---

### CONNECTICUT VALLEY DENTAL SOCIETY.

The semi-annual meeting of the Connecticut Valley Dental Society will be held at the Sea View House, Savin Rock, New Haven, Connecticut, Wednesday and Thursday, June 18 and 19, 1884.

W. F. ANDREWS, Secretary.

### Askings and Answers.

So many questions upon professional subjects have been at different times sent to us, that it has been deemed advisable to open a special department for their consideration. We shall be glad if those desiring information will forward any pertinent questions, and if those possessed of the desired knowledge will send concise answers. In the absence of volunteer replies, we shall refer any important subjects to such of our associates or friends as are possessed of special knowledge of the matter enquired about.

---

If you will kindly give me room in your valuable Journal, I would like to ask a question of some of your subscribers who are better posted than I am.

Several times I have removed old leaky amalgam fillings and found a mass of black, pasty matter underneath. This I have carefully washed out, cut away the decay, cleansed and disinfected the root canals as thoroughly as possible, and then refilled roots and cavity as nicely as I was able—and then after all the care and labor bestowed upon them, have had my patients return next day suffering severe pain, and the teeth so treated elongated and sore to the touch! Now why should these teeth which the patients declared never gave them trouble before, even when loaded with decomposed matter, give such trouble when cleaned and refilled? J. L. D.

---

Will somebody please inform me in the next number of the INDEPENDENT PRACTITIONER, what difference (if any) it makes, what sort of filling is employed for closing up pulp canals? A. B. L., Kansas.

*Answer.*—See editorial in this number. Ed.

---

When excavating large cavities in teeth preparatory to filling, we sometimes find the tooth-pulp nearly exposed, and protected only by a thin layer of dentine, much discolored and not very dense. Now, is it better in these cases to cut out all this discolored portion and risk exposing the tooth-pulp, or leave it and fill over it? B——.

---

I have seen several cases in which dental periostitis, which had resisted the usual local treatment, was promptly cured (so the patients said) by homeopathic medicines. Can anybody give the particulars of such treatment? J. S.

---

Calcium Sulphide is used in general medicine as a preventive and cure for periostitis. Has it any record in dental practice? D.

*Answer.*—Dr. Geo. B. Snow not long since read a paper before the Buffalo Dental Association, in which he strongly recommended this remedy in case of acute alveolar abscess, and recited instances of its successful employment. The paper may be found in the April number of *The Dental Advertiser*. Ed.

